# Fluconazole-resistant *Candida parapsilosis*: A new emerging threat in the fungi arena

**DOI:** 10.3389/ffunb.2022.1010782

**Published:** 2022-10-24

**Authors:** Pilar Escribano, Jesús Guinea

**Affiliations:** ^1^ Clinical Microbiology and Infectious Diseases, Hospital General Universitario Gregorio Marañón, Madrid, Spain; ^2^ Instituto de Investigación Sanitaria Gregorio Marañón, Madrid, Spain; ^3^ CIBER Enfermedades Respiratorias-CIBERES (CB06/06/0058), Madrid, Spain

**Keywords:** *Candida parapsilosis*, fluconazole, resistance, ERG11, Y132F, G458S, K128N, K143R

## Abstract

*Candida parapsilosis* is a leading cause of invasive candidiasis in southern Europe, Latin America and Asia. *C. parapsilosis* has been mostly considered susceptible to triazoles, but fluconazole resistance is on the rise in some countries. The main mechanism related to fluconazole resistance is the presence of ERG11p substitutions, dominated by the Y132F amino acid substitution. Isolates harbouring this substitution mimic *C. auris* given that they may cause hospital outbreaks, become endemic, and emerge simultaneously in distant areas around the world. At the moment, Spain is experiencing a brusque emergence of fluconazole resistance in *C. parapsilosis*; isolates harbouring the Y132F substitution were detected for the first time in 2019. A recent study on *Candida* spp isolates from blood cultures collected in 16 hospitals located in the Madrid metropolitan area (2019 to 2021) reported that fluconazole resistance in *C. parapsilosis* reached as high as 13.6%. Resistance rates rose significantly during those three years: 3.8% in 2019, 5.7% in 2020, and 29.1% in 2021; resistant isolates harboured either the dominant Y132F substitution (a single clone found in four hospitals) or G458S (another clone found in a fifth hospital). The COVID-19 pandemic may have increased the number of candidaemia cases. The reason for such an increase might be a consequence of uncontrolled intra-hospital patient-to-patient transmission in some hospitals, as an increase not only in *C. parapsilosis* candidaemia episodes but also in the spread of clonal fluconazole-resistant isolates might have occurred in other hospitals during the pandemic period. Patients affected with fluconazole-resistant *C. parapsilosis* harbouring the Y132F substitution presented a mortality rate ranging from 9% to 78%, were mainly admitted to intensive care wards but did not have differential risk factors compared to those infected by susceptible isolates. With scarce exceptions, few patients (≤20%) infected with fluconazole-resistant isolates had previously received fluconazole, thus supporting the fact that, although fluconazole might have been a key factor to promote resistance, the main driver promoting the spread of fluconazole-resistant isolates was patient-to-patient transmission.

## Introduction

*Candida parapsilosis* is a leading cause of invasive candidiasis, particularly in southern Europe, Latin America and Asia (Nucci et al., 2010; Guinea et al., 2014; Guo et al., 2021). A population-based study conducted in Spain showed that *C. parapsilosis* accounted for over 25% of all candidaemia isolates collected (Guinea, 2014). A single-hospital study confirmed a similar and steady-over-time proportion of episodes of candidaemia caused by *C. parapsilosis* in patients admitted to a hospital located in Madrid from 2007 to 2019; the same observation was recently confirmed in a multi-centre study involving 16 hospitals located in the same city (Diaz-Garcia et al., 2021b, Díaz-García et al., 2022b). The reasons why *C. parapsilosis* is a frequent cause of candidaemia in some geographic areas is still unknown. Since *C. parapsilosis* represented 27% of isolates from blood cultures but only 2.6% from intra-abdominal samples (Diaz-Garcia et al., 2021a), the species seems to be prone to cause catheter-related candidaemia (Puig-Asensio et al., 2014).

*C. parapsilosis* has been mostly considered susceptible to fluconazole and newer triazoles, however, the number of reported fluconazole-resistant isolates has been increasing simultaneously in different countries in the last few years. Azole resistance in *C. parapsilosis* is a matter of concern given the intrinsic diminished susceptibility of the species to echinocandins (Diaz-Garcia et al., 2021b). The environmental trait of *C. parapsilosis*, its high ability to form biofilms, and its potential to promote fluconazole resistance may explain the spread of fluconazole-resistant isolates across some hospitals, to the point of becoming endemic and a public health problem.

The present review covers the state-of-the-art of the emerging threat concerning fluconazole resistance in *C. parapsilosis*. The topics revised include an overview of the fluconazole resistance rates in *C. parapsilosis*, the dominant underlying mechanisms of resistance, the spread and tracking of resistant isolates, and the clinical impact of resistance. We performed a literature search in PubMed using the following keywords: *“Candida*”, “*parapsilosis*”, “fluconazole”, and “resistance”. Of the studies found (n=884), for the current review, we selected those reporting fluconazole antifungal susceptibility data, characterization of resistance mechanisms, and genotyping (n=38).

## Rates of fluconazole resistance in *C. parapsilosis*

*C. parapsilosis* has been historically reported as susceptible to fluconazole; however, several reports have warned of the recent increasing rate of fluconazole resistance in some geographic regions. A study conducted on isolates collected worldwide between 1997 and 2016 reported a fluconazole resistance rate of 3.9% (Pfaller et al., 2019). Likewise, a population-based study conducted in Spain in 2010 and 2011 reported a low rate of fluconazole resistance (2.5%) in that species (Guinea et al., 2014). Other studies reported similar rates in Italy (3.6%) (Prigitano et al., 2016), Portugal (4%) (Faria-Ramos et al., 2014), and Denmark (6%) (Arendrup et al., 2011).

However, multi-centre studies may overshadow local epidemiology. For example, high azole resistance rates have been reported in single-centre studies recently conducted in France (9.2%) (Fekkar et al., 2021), Turkey (26.4%) (Arastehfar et al., 2020a), Italy (33%) (Mesini et al., 2020), Saudi Arabia (33%) (Aldardeer et al., 2020), Mexico (54%) (Corzo-Leon et al., 2021), Brazil (67.9%) (Thomaz et al., 2021), and South Africa (78%) (Magobo et al., 2020). Local epidemiology among hospitals located in the same area may differ. For example, fluconazole-resistant *C. parapsilosis* was not detected in patients admitted to the Gregorio Marañón hospital (Madrid, Spain), whereas other hospitals located in the same region reported disparate resistance rates (Diaz-Garcia et al., 2021a, Diaz-Garcia et al., 2021b, Diaz-Garcia et al., 2022b). Such notable differences among centres can be the consequence of infection control policies, prior use of azoles, and the fluconazole-resistant clones spreading across hospitals.

## Molecular basis of fluconazole resistance in *C. parapsilosis*

Fluconazole prevents fungal *Candida* spp cell growth by inhibiting lanosterol 14-α demethylase (ERG11p), a protein encoded by the *ERG11* gene, which leads to a blockade of ergosterol synthesis, an essential component of fungal cell membranes (Grossman et al., 2015). Fluconazole resistance mechanisms are well known in *C. albicans* and their understanding has been helpful to bridge the knowledge gap in species such as *C. parapsilosis*.

Fluconazole resistance mechanisms may work alone or simultaneously. A major mechanism of resistance in *C. albicans* involves the *ERG11* gene, in which the presence of some substitutions may lead to either a reduced affinity of ERG11p for the drug (Sanglard and Odds, 2002) or *ERG11* gene up-regulation when mutations occur in the *UPC2* gene encoding the transcriptional regulator of sterol biosynthesis genes (Schneider and Morschhauser, 2015). Some substitutions in the *ERG3* gene (encoding a sterol-desaturase) may lead to loss of function of the enzyme, promote the accumulation of deleterious sterols, and allow the fungus to survive in the presence of fluconazole; however, these mechanisms are much less frequent (Morio et al., 2012). The other main mechanism involves the presence of mutations in the *TAC1* (transcriptional activator of *CDR* genes) and *MRR1* (transcription factor and multidrug resistance regulator) genes, which lead to overexpression of *CDR* and *MDR1*, respectively, resulting in the pumping of fluconazole out of the cell (Morschhauser et al., 2007; Liu and Myers, 2017).

**Figure 1:**
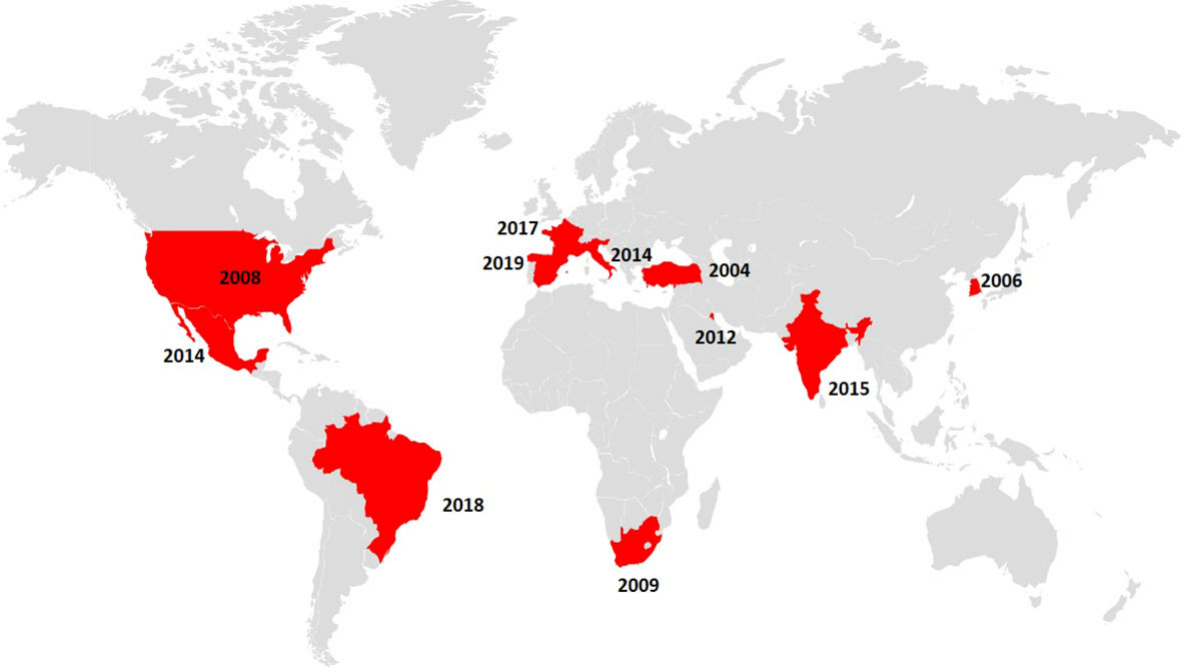
Map indicating the countries in which fluconazole-resistant *C. parapsilosis* isolates harbouring the Y132F ERG11p substitution were reported (depicted in red) alongside the year of detection of the first isolate.

The dominant mechanism of fluconazole resistance in *C. parapsilosis* is the presence of ERG11p substitutions (**Table 1**). Y132F is the dominant amino acid substitution and has also been described in *C. albicans*, *C. tropicalis*, and *C. auris* (Morio et al., 2010; Jiang et al., 2013; Healey et al., 2018). *C. parapsilosis* isolates harbouring the Y132F ERG11p substitution have been recently reported in several countries (**Table 2** and **Figure 1**). The first isolate was detected in Turkey in 2004 followed by other countries, with Spain being the last to be added to the list in 2019. The number of affected countries is expected to increase in the near future.

**Table 1:** ERG11p substitutions reported in *C. parapsilosis* isolates and their fluconazole resistance profile.

ERG11p amino acid substitutions	Expected pattern of azole susceptibility		References
		Fluconazole		Voriconazole					
Y132F*	R	R or I	(Grossman et al., 2015; Souza et al., 2015; Asadzadeh et al., 2017; Magobo et al., 2017; Choi et al., 2018; Thomaz et al., 2018; Alobaid and Khan, 2019; Singh et al., 2019, Arastehfar et al., 2020a, Arastehfar et al., 2020b, Castanheira et al., 2020; Magobo et al., 2020; Martini et al., 2020; Arastehfar et al., 2021; Corzo-Leon et al., 2021; Demirci-Duarte et al., 2021; Fekkar et al., 2021; Thomaz et al., 2021; Alcoceba et al., 2022, Diaz-Garcia et al., 2022a, Hare et al., 2022; Thomaz et al., 2022; Zaragoza et al., 2022)
K143R**	R	S	(Singh et al., 2019, Arastehfar et al., 2020a, Castanheira et al., 2020)
G458S***	R	R or I	(Arastehfar et al., 2020a, Demirci-Duarte et al., 2021, Diaz-Garcia et al., 2022a)
**K128N**	R	S	(Choi et al., 2018)
D133Y	S	S	(Hare et al., 2022)
D247G	S	S	(Arastehfar et al., 2020b)
G89V	S	S	(Arastehfar et al., 2020b)
I302T	S	S	(Hare et al., 2022)
L109F	S	S	(Arastehfar et al., 2020b)
M178T	SDD or S	S	(Grossman et al., 2015; Asadzadeh et al., 2017)
N283Y	SDD or S	S	(Grossman et al., 2015; Asadzadeh et al., 2017)
P406Q	S	S	(Arastehfar et al., 2020b)

R, resistant; S, susceptible; I, intermediate; SDD, susceptible dose-dependent.

Substitutions in bold have been associated with fluconazole resistance.

*Y132F substitution may be present in combination with substitutions K143R, D421N, G307A, and R398I. Very few isolates harbouring the Y132F substitution were reported as fluconazole susceptible (Castanheira et al., 2020), fluconazole-intermediate (Demirci-Duarte et al., 2021), or voriconazole-susceptible (Grossman et al., 2015).

**The K143R substitution may be present in combination with Y132F substitutions. Very few isolates harbouring the K143R substitution were reported as fluconazole-intermediate (Castanheira et al., 2020), or voriconazole-resistant (Arastehfar et al., 2020a).

***The G458S substitution may be present in combination with substitutions Q250K, and T519A. Very few isolates harbouring the G458S substitution were reported as voriconazole-intermediate (Arastehfar et al., 2020a) or voriconazole-susceptible (Demirci-Duarte et al., 2021).

**Table 2:** Studies in which fluconazole-resistant *C. parapsilosis* isolates harbouring Y132F, K143R, G458S, and K128N ERG11p substitutions were reported for the first time (year of detection) in the countries affected.

Country	ERG11p substitution(Year of first isolate detection)	Year of publication	Reference
Turkey	Y132F (2004)	2021	(Demirci-Duarte et al., 2021)
	G458S (2011)		
	K143R (2011)	2020	(Arastehfar et al., 2020a)
South Korea	Y132F (2006)	2018	(Choi et al., 2018)
	K128 N (2015)		
USA	Y132F (2008)	2015	(Grossman et al., 2015)
	K143R (2017)	2020	(Castanheira et al., 2020)
South Africa	Y132F (2009)	2020	(Magobo et al., 2020)
Kuwait	Y132F (2012)	2017	(Asadzadeh et al., 2017)
Mexico	Y132F (2014)	2021	(Corzo-Leon et al., 2021)
Italy	Y132F (2014)	2020	(Martini et al., 2020)
India	K143R (2015)	2019	(Singh et al., 2019)
	Y132F (2015)		
France	Y132F (2017)	2020	(Castanheira et al., 2020)
Brazil	Y132F (2018)	2018	(Thomaz et al., 2018)
Spain	Y132F (2019)	2022	(Alcoceba et al., 2022)
	K143R (2020)	2022	(Zaragoza et al., 2022)
	G458S (2021)	2022	(Diaz-Garcia et al., 2022a)

The presence of G458S, K128N, and K143R ERG11p substitutions has been also associated with fluconazole resistance (**Table 1**). Substitutions can sometimes be found in combination; among others, the R398I substitution, as well as silent mutations, have been previously described in both susceptible and resistant isolates. Therefore, the role of these “accompanying” or “compensatory” mutations is uncertain and may represent mere polymorphisms, not necessarily associated with resistance (**Table 1**) (Grossman et al., 2015; Singh et al., 2019).

The profile of azole resistance is influenced by the type of amino acid substitution, although the data available is very limited. Isolates harbouring ERG11p substitutions are fully susceptible to amphotericin, micafungin and anidulafungin, and ibrexafungerp also (Diaz-Garcia et al., 2022b). Isolates harbouring the Y132F substitution are fluconazole-resistant and the vast majority are either voriconazole-resistant or voriconazole-intermediate (**Table 1**); one study reported that some isolates could also be non-wild type to posaconazole or isavuconazole (Diaz-Garcia et al., 2022a). Isolates harbouring the G458S substitution are fluconazole-resistant and mostly voriconazole-resistant (**Table 1**); one study reported that all isolates were non-wild type to posaconazole or isavuconazole, thus suggesting a pan-azole resistance phenotype (**Table 1**) (Diaz-Garcia et al., 2022a). Isolates with the K143R substitution are fluconazole-resistant and mostly voriconazole-susceptible and posaconazole and isavuconazole wild type (**Table 1**). Finally, isolates with the K128N substitution were reported in a single study as fluconazole-resistant and voriconazole-susceptible (**Table 1**).

Some fluconazole-resistant *C. parapsilosis* isolates presented a wild-type *ERG11* gene sequence (Arastehfar et al., 2020a, Arastehfar et al., 2020b), thus suggesting alternative mechanisms of resistance. At this point in time, such alternative mechanisms play an uncertain role in azole resistance. Missense mutations in the transcriptional regulators *TAC1* and *MRR1* do not necessarily correlate with the overexpression of *CDR1* and *MDR1* (Berkow et al., 2015; Grossman et al., 2015; Asadzadeh et al., 2021). The genome plasticity and aneuploidy of *C. parapsilosis* can promote cell growth in the presence of azoles and have been suggested as a resistance mechanism (Yang et al., 2021).

## Spread of fluconazole-resistant *C. parapsilosis* harbouring ERG11p substitutions

*Candida* spp can commonly cause outbreaks of candidaemia in patients admitted to intensive care units and post-surgical units (Guducuoglu et al., 2016; Guinea et al., 2021). *C. parapsilosis* outbreaks have been reported in adult/neonatal intensive care units, frequently associated with patient-to-patient transmission (Clark et al., 2004; Diab-Elschahawi et al., 2012). In the event of an outbreak, the study of infecting isolates and environmental isolates, collected from the vicinity of the patient, is needed to abate the outbreak.

Genotyping of isolates using highly discriminative tools may be useful to unravel the outbreak source and support infection control investigations. This approach might be particularly useful in hospital wards with a high number of candidaemia episodes. A variety of methods are available for genotyping, such as Southern blot hybridization with specific probes, electrophoretic karyotyping, amplified fragment length polymorphism (AFLP) analysis, restriction fragment length polymorphism (RFLP) analysis, randomly amplified polymorphism DNA (RAPD) analysis, multilocus sequence typing, and microsatellites, however, there is no ‘gold standard’ as yet (Marcos-Zambrano et al., 2014). Microsatellites are an attractive method and species-specific panels have been developed for *C. parapsilosis* (Lasker et al., 2006; Sabino et al., 2010; Diab-Elschahawi et al., 2012).

We have been genotyping *Candida* spp isolates from blood cultures for the last 20 years; some genotypes may be found only in a given patient (singleton genotypes) whereas others may be found in two or more patients (clusters) (Guinea et al., 2020). The presence of clusters could suggest a common source of infection or patient-to-patient transmission and cause infections in the form of outbreaks. Clusters may go unnoticed and are only unveiled by blindly genotyping consecutive isolates causing candidaemia (Escribano et al., 2013). A high number of clusters could indicate high patient-to-patient transmission in hospital wards with a high incidence of candidaemia; as a matter of fact, the implementation of prevention campaigns regarding catheter-related infections in our hospital correlated with a decrease in both the number of candidaemia episodes and the number of *C. albicans* and *C. parapsilosis* clusters (Escribano et al., 2018).

We previously observed that the presence of *C. albicans* and *C. parapsilosis* clusters was not infrequent and both species fulfilled a species-specific pattern: while *C. albicans* clusters involved a limited number of patients and were limited in time, *C. parapsilosis* clusters involved a higher number of patients, could become endemic in the unit, and persist in the ward for years (Guinea et al., 2021). Not all clusters involved patients with an epidemiological relationship; some patients involved in a given cluster could be admitted to different hospital wards at the same hospital or even different hospitals (Escribano et al., 2018; Guinea et al., 2020). The reason for these “unexplained” clusters is unclear but they may represent genotypes prone to being widespread across different geographic regions. When isolates are tagged by a phenotypic characteristic, such as the presence of fluconazole resistance, isolate genotyping may be helpful to track them down and understand the dynamics of their transmission, as well as understanding the clones implicated.

Several studies have shown the ability of *C. parapsilosis* isolates mainly harbouring substitution Y132F ERG11p to cause hospital outbreaks (Grossman et al., 2015; Souza et al., 2015; Pinhati et al., 2016; Choi et al., 2018; Thomaz et al., 2018; Singh et al., 2019; Magobo et al., 2020; Martini et al., 2020; Arastehfar et al., 2021; Corzo-Leon et al., 2021; Fekkar et al., 2021; Thomaz et al., 2021). The clonal spread of fluconazole-resistant isolates is not surprising and follows the previously described pattern of *C. parapsilosis* isolates in the clinical setting. Some clusters harbouring the Y132F substitution became endemic in the hospital environment (Choi et al., 2018; Thomaz et al., 2018; Martini et al., 2020; Alcoceba et al., 2022; Ramos-Martinez et al., 2022). Sometimes, different *C. parapsilosis* genotypes harbouring the Y132F substitution coexisted within a particular hospital (Magobo et al., 2017; Choi et al., 2018; Arastehfar et al., 2021; Corzo-Leon et al., 2021; Thomaz et al., 2021). Conversely, a particular clone could be found in different hospitals (Grossman et al., 2015; Magobo et al., 2017; Zhang et al., 2020, Diaz-Garcia et al., 2022a, Thomaz et al., 2022). These observations may simply reflect transfers of patients among hospitals of a given region and the active spread of isolates across hospitals (Chow et al., 2018). Isolates harbouring the Y132F substitution mimic *C. auris* given that they may cause hospital outbreaks, become endemic, and emerge simultaneously in distant areas around the world.

Studies reporting isolates harbouring the remaining relevant ERG11p substitutions (K134R, G458S, and K128N) are very limited. One study reported a number of isolates harbouring the K134R substitution in patients admitted to Indian hospitals. These isolates followed a pattern similar to that found in isolates harbouring the Y132F substitution: different clones could be found in a given hospital, whereas a clone could be found in different hospitals (Singh et al., 2019). We recently conducted a surveillance study in the Madrid metropolitan area in which we reported the presence of isolates harbouring the G458S substitution in a single hospital (Diaz-Garcia et al., 2022a). Finally, to date, isolates harbouring the K128N substitution have not been reported as a cause of outbreaks (Choi et al., 2018).

A major limitation of previously reported studies lies in the fact that they were mostly conducted retrospectively. Furthermore, environmental isolates were only genotyped in a single study conducted in Brazil, which proved the presence of resistant isolates in the vicinity of the patients (Thomaz et al., 2021). Although limited, available data suggests that the niche of fluconazole-resistant *C. parapsilosis* isolates may be in the environment of the infected patients.

## Emergence of fluconazole-resistant *C. parapsilosis* isolates in Spain

Spain is currently experiencing a brusque emergence of fluconazole resistance in *C. parapsilosis*. The rate of fluconazole resistance in *C. parapsilosis* reported in the CANDIPOP study conducted in Spain in 2010 and 2011 was 2.5% (Guinea et al., 2014). Ten years later, the presence of fluconazole-resistant *C. parapsilosis* isolates harbouring the Y132F substitution was reported for the first time in Spain, in isolates collected in 2019 in the Son Espases hospital, a public 750-bed tertiary referral hospital located in the Balearic Islands (Alcoceba et al., 2022). The fact that the first resistant isolate (fluconazole plastic strip MIC > 4 mg/L) was detected in October 2015 (isolates collected from 2015 to April 2019 were unfortunately unavailable) suggested that the onset of the outbreak was in 2015 rather than in 2019. Since then, the number of patients with fluconazole-resistant *C. parapsilosis* isolates has been on the rise and fluconazole resistance rates in *C. parapsilosis* overall (83.7%) or isolates from blood cultures (70%) were extremely high.

The Son Espases hospital study represented a textbook example of a hospital severely hit by fluconazole-resistant *C. parapsilosis* (Alcoceba et al., 2022). The first resistant isolate showed up a long time ago, then its offspring adapted to the hospital environment to the point of becoming endemic. Such a spread may have been a consequence of delayed detection of resistant isolates; for this reason, prospective and long-lasting antifungal resistance surveillance studies are helpful in terms of allowing the early detection of resistant isolates and monitoring the resistance trend. In January 2019, we initiated the prospective monitoring of antifungal resistance in *Candida* spp isolates collected from blood cultures and intra-abdominal samples of patients admitted to any of the 16 hospitals located in the Madrid metropolitan area (CANDIMAD study). Data on 2,107 *Candida* spp isolates (1,895 patients) collected from 2019 to 2021 in the CANDIMAD study were recently reported (Diaz-Garcia et al., 2022b), including a separate analysis of *C. parapsilosis* (Diaz-Garcia et al., 2022a). Fluconazole resistance was higher in blood cultures than in intra-abdominal samples (9.1% versus 8.2%; P>0.05), especially for *C. parapsilosis* (16.6% versus 3.6%, P<0.05). A total of 13.6% of *C. parapsilosis* isolates were fluconazole-resistant and sourced mostly from blood cultures (94%). Moreover, overall resistance rates in *C. parapsilosis* were rising during these three years: 3.8% in 2019, 5.7% in 2020, and 29.1% in 2021 (*P*<0.05). *C. parapsilosis* resistant isolates involved patients admitted to five hospitals and were detected for the first time in May 2019, September 2020, October 2020, February 2021, and August 2021, at each hospital. Hospitals were affected to a different extent; whereas the fluconazole resistance rate in some hospitals was 0%, it reached up to 37.7% in others. All fluconazole-resistant isolates harboured either the Y132F (n=43/48) or G458S (n=5/48) substitution in ERG11p. Isolates harbouring the Y132F substitution were found in four hospitals, whereas isolates harbouring the G458S substitution were found in the fifth hospital.

All fluconazole-resistant isolates collected at the Son Espases hospital grouped into 11 clonally related genotypes, with one genotype accounting for most isolates. Likewise, all fluconazole-resistant isolates collected in the Madrid surveillance study harbouring the Y132F substitution grouped into three clonally related genotypes; one genotype dominated and was the first detected, whereas isolates harbouring the G458S substitution grouped into another genotype. On balance, the fluconazole-resistant genotypes detected in the Balearic Islands and Madrid were different one from another, and different from those involving susceptible isolates. To date, two main observations can be drawn. First, clones harbouring the Y132F substitution in the two regions of the country were different and caused unrelated outbreaks. Second, the clone harbouring the G458S substitution in Madrid was different and emerged independently in another hospital. At the moment, we are tracking down fluconazole-resistant *C. parapsilosis* isolates from other Spanish regions and countries. Moreover, we are keeping a closer watch on the evolution of the resistant clones in the Madrid region.

## Fluconazole-resistant *C. parapsilosis* isolates and the COVID-19 pandemic

The COVID-19 pandemic may have increased the number of cases of candidaemia (Nucci et al., 2021; Machado et al., 2022; Papadimitriou-Olivgeris et al., 2022; Seagle et al., 2022). This increase may be a consequence of either an increase in the number of at-risk patients admitted or higher patient-to-patient transmission, but this might be a hospital-dependent phenomenon. In our hospital, whereas the incidence of candidaemia was higher in patients with COVID-19 than without, genotyping demonstrated that the increase was not due to uncontrolled intra-hospital patient-to-patient transmission (Machado et al., 2022). Conversely, a study conducted in another Madrid hospital showed that *C. parapsilosis* candidaemia episodes increased significantly during the pandemic period, including the number of cases caused by a fluconazole-resistant *C. parapsilosis* genotype harbouring the Y132F ERG11p substitution and spreading at that time (Ramos-Martinez et al., 2022). In line with these observations, another study conducted in Greece showed that both the incidence of candidaemia and fluconazole resistance in *C. parapsilosis* increased during the pandemic; unfortunately, the isolates were not molecular characterized (Routsi et al., 2022). In the Madrid study, many resistant isolates showed up before the COVID-19 pandemic; however, their spread become more evident during the pandemic, suggesting that all triggers increasing the number of candidaemia cases may have been effective in terms of promoting the clonal spread of resistant isolates (Ramos-Martinez et al., 2022).

## Clinical impact of infections caused by fluconazole-resistant *C. parapsilosis* isolates harbouring the Y132F ERG11p substitution

The clinical description of patients affected with fluconazole-resistant *C. parapsilosis* harbouring the Y132F ERG11p substitution is quite limited. Some studies compared patients infected with fluconazole-resistant isolates with those infected with susceptible ones and did not find any differences in terms of clinical presentation or risk factors (Fekkar et al., 2021; Alcoceba et al., 2022). The reports warn that a large number of patients affected with resistant isolates were admitted to intensive care wards (Thomaz et al., 2018; Corzo-Leon et al., 2021; Fekkar et al., 2021; Thomaz et al., 2021; Alcoceba et al., 2022; Ramos-Martinez et al., 2022). An interesting observation was the fact that, with an exception (Thomaz et al., 2018), few patients infected with fluconazole-resistant isolates had previously received fluconazole (≤20%) (Corzo-Leon et al., 2021; Fekkar et al., 2021; Alcoceba et al., 2022; Ramos-Martinez et al., 2022). This supports the fact that, fluconazole might have been a key factor in promoting resistance, however, the main driver promoting the spread of fluconazole-resistant isolates was patient-to-patient transmission. Patient mortality ranged from 9% to 78% (Thomaz et al., 2018; Corzo-Leon et al., 2021; Fekkar et al., 2021; Thomaz et al., 2021; Alcoceba et al., 2022; Ramos-Martinez et al., 2022).

This review demonstrates the importance of tracking, genotyping, and controlling the spread of fluconazole-resistant *C. parapsilosis* isolates. Future studies are warranted in order to assess the clinical impact of this emerging problem, the quality of hospital control policies to halt future spreading, as well as the development of techniques to allow a fast and accurate detection of isolates in routine clinical microbiology.

## Author contributions

PE and JG wrote all sections of the manuscript. Both authors contributed to the article and approved the submitted version.

## Funding

This work was supported by grants PI18/01155 and PI19/00074 from the *Fondo de Investigación Sanitaria* (*FIS*. *Instituto de Salud Carlos III. Plan Nacional de I+D+I* 2017-2020). The study was co-funded by the European Regional Development Fund (FEDER) ‘A way of making Europe.’ The funders had no role in the study design, data collection, analysis, decision to publish, or preparation/content of the manuscript. PE (CPI20/00015) is a recipient of a Miguel Servet contract supported by the *FIS*. JG is a permanent researcher contracted by *Fundación para Investigación Sanitaria del Hospital Gregorio Marañón*.

## Acknowledgments

We are grateful to Helena Kruyer for editing assistance.

## Conflict of interest

JG has received funds for participating in educational activities organized on behalf of Pfizer, Gilead, and MSD; he has also received research funds from FIS, Gilead, Scynexis, F2G, and Cidara outside the submitted work.

The remaining author declares that the research was conducted in the absence of any commercial or financial relationships that could be constructed as a potential conflict of interest.

## Publisher’s note

All claims expressed in this article are solely those of the authors and do not necessarily represent those of their affiliated organizations, or those of the publisher, the editors and the reviewers. Any product that may be evaluated in this article, or claim that may be made by its manufacturer, is not guaranteed or endorsed by the publisher.
